# What Makes People Hide Knowledge? Influence of Passive Leadership and Creative Self-Efficacy

**DOI:** 10.3389/fpsyg.2021.740880

**Published:** 2021-10-08

**Authors:** Namra Mubarak, Atasya Osmadi, Jabran Khan, Amir Mahdiyar, Asim Riaz

**Affiliations:** ^1^School of Housing Building and Planning, Universiti Sains Malaysia, Penang, Malaysia; ^2^Air University School of Management, Air University, Islamabad, Pakistan; ^3^Department of Management Sciences, Capital University of Science and Technology, Islamabad, Pakistan

**Keywords:** knowledge hiding, passive leadership, creative self-efficacy, projects, knowledge

## Abstract

Although numerous studies have been conducted in the field of knowledge sharing with a focus given to its importance, very little attention has been given to knowledge hiding practices. A very few studies have been found to make an attempt to figure out its impact and antecedents. Likewise, the negative role of passive leadership in the project management literature has not been evidenced enough despite its existence in project-based organizations. Both knowledge hiding and passive leadership are the highly neglected areas in the project management literature. Therefore, this study not only attempts to investigate the influence of passive leadership on knowledge hiding but also aims to explore the role of creative self-efficacy between them. IT project organizations were chosen to collect data because of their high failure rate due to an insufficient knowledge transfer. The findings of this study revealed that the neglected passive leadership greatly influences the knowledge hiding practices among individuals. However, according to the results, knowledge hiding practices are found to reduce the presence of creative self-efficacy. Thus, the antecedents of knowledge hiding should be considered to create an innovative and successful business environment. The results are highly significant not only for the field of project management but also for other practitioners.

## Introduction

A few studies show that the exchange of information is vital for the successful completion of tasks in project-based organizations (Singh, 2019). Although researchers have been highlighting the importance of knowledge sharing (Ruuska and Vartiainen, 2005; Cerne et al., 2014; Škerlavaj et al., 2018), the issue of knowledge hiding behavior has largely been ignored. It has been observed that more than 75% of individuals hide information from their fellow workers (The Globe and Mail, 2006). Hiding is an intentional attempt to hold knowledge (Connelly et al., 2012). The knowledge hiding practice accelerates when a person feels psychological ownership of the knowledge (Hernaus et al., 2019). Even if organizations motivate employees to share knowledge, they still indulge in knowledge hiding practices (Connelly et al., 2012). This ultimately results in hindering the knowledge that is essential for completing the project tasks. It is also important to say that knowledge hiding is an individually targeted behavior because it pertains to the intentional denial of knowledge requests from the requestors (Pan et al., 2018). Knowledge hiding can most likely affect the outcomes, and therefore it is very important to understand the roots of this behavior to eliminate it.

The antecedents of knowledge hiding have also been the subject matter of recent research. Among many other possible antecedents, a few studies also identify that bad leadership style and behavior show disengagement toward employees and the workplace. Moreover, few studies indicate that passive leaders neither do take necessary action nor do not communicate timely (Barling and Frone, 2017). One of the possible traits of passive leadership includes knowledge hiding. It has been observed that passive leadership encourages knowledge hiding behaviors as they do not participate and engage in passing the information in time (Irum et al., 2020). The dyadic interactions at the workplace are governed by an unspoken social exchange between coworkers (Blau, 1964), which may be the result of passive leadership. However, discouraging passive leadership might result in better social exchange (Vullinghs et al., 2018). Poor social exchange by a passive leader will result in knowledge hiding behavior. A good leader not only develops knowledge content required for employees but also helps in its adoption by sharing and facilitating (Chase et al., 2020), which is not the trait of a passive leader. The interaction between leaders and subordinates is very important; however, most of the time, knowledge hiding behavior is observed extensively (Connelly et al., 2012; Peng, 2013; Cerne et al., 2014; Connelly and Zweig, 2015). Despite its presence in many projects, the impact of knowledge hiding practices in projects due to passive leadership is yet to be explored (Ladan et al., 2017; Fong and Slotta, 2018; Butt and Ahmad, 2019). There is a dire need to explore this area because it is an assumption that passive leadership may promote knowledge hiding practices (Chen, 2020). This is because of the fact that the personal attitude of an individual gets influenced by subjective norms (Ajzen, 1991). Subjective norms, i.e., passive leadership, not only limit social exchange but also limit the interactions, collaborations, and motivation (Chênevert et al., 2015), possibly resulting in knowledge hiding practices. Hence, passive leadership is certainly linked to knowledge hiding. Both knowledge hiding and passive leadership have been given very less attention in social sciences.

There are many factors that may reduce the effects of negative leadership style, e.g., personal resources (De Clercq and Belausteguigoitia, 2017), including creative self-efficacy, personality, mindfulness, hardiness, and psychological capital (Xanthopoulou et al., 2007). According to Arain et al. (2019), negative leadership style is influenced by the creative self-efficacy of employees. Passive leadership is part of a negative leadership style wherein leaders act inactive with very limited interaction (Barling and Frone, 2017). Reduced communication by the leader impacts the information-sharing capability of individuals (Yang et al., 2017). Thus, a very limited collaboration results in more complexities. However, if employees have creative self-efficacy, then it will not only improve their wellbeing but also motivate them to surpass difficulties and avoid hindrances (Bandura, 1997). Individuals with creative self-efficacy are motivated to complete the required tasks by working together and sharing knowledge (Hu and Zhao, 2016). Therefore, knowledge sharing comes with motivation (Hendriks, 1999) as it is the case of creative self-efficacy of employees. This phenomenon also results in reducing the knowledge hiding behavior of employees.

Putting this in other words, passive leadership results in poor communication, disengagement, and knowledge hiding behavior. However, if employees involve creative self-efficacy, then they will be able to share knowledge and produce something creative by working with each other. Thus, the negative effects of passive leadership resulting in knowledge hiding can be alleviated through the creative self-efficacy of employees. This personal resource of employees is very important to lessen the knowledge hiding behavior. Similarly, another study links abusive supervision to the knowledge hiding behavior of employees (Cerne et al., 2017). One more study also reports how self-efficacy mitigates the influence of negative leadership resulting in low knowledge hiding behavior (Arain et al., 2019). However, creative self-efficacy has not yet been studied in relationship with the passive leadership style, i.e., negative leadership style and knowledge hiding behavior.

## Theory and Hypotheses

The model for this study derives from the theory of planned behavior: personal attitude (whether I want to do it or not), subjective norms (do other individuals want me to do it), and perceived behavioral control (whether I have the ability to do that) (Ham et al., 2015). Knowledge hiding is the personal attitude formulated by a subjective norm and behavioral control. On one hand, passive leadership acts as a subjective norm in such a way that it influences the knowledge-sharing behavior of other individuals, due to which they hide knowledge. On the other hand, behavioral control (creative self-efficacy) acts as perceived behavioral control and is the ability of an individual to be creative and sharing even in the presence of passiveness—it shows that how much behavioral control one has even in the presence of negativity (Ajzen, 1991). According to the theory of planned behavior, behavioral control triggers the intention, i.e., whether to hide knowledge or not. Moreover, according to the theory of planned behavior, behavioral intention, along with perceived behavioral control, can be directly used to forecast behavioral achievement, i.e., project success (Ajzen, 1991). The scarcity of studies identifying the antecedents of knowledge hiding aggravates the need to address the issue of knowledge hiding. Thus, based on the theory of planned behavior, this study not only highlights the role of leadership (i.e., passive leadership) toward knowledge hiding, but also the impact of personal resources (i.e., creative self-efficacy) as a moderator between knowledge hiding and passive leadership.

### Knowledge Hiding and Passive Leadership

Knowledge hiding has been seen to cause an immense loss. It has also been seen to impact some of the largest economies of the world, i.e., 46% of Chinese workers and 76% of the US workers tend to show knowledge hiding behavior (Pan et al., 2018). This behavior is not only limited to the developed economies; a mean score of 2.9 of 5 is reported in Pakistani workers who hide knowledge (Khalid et al., 2018). Similar trends have been observed in other emerging economies. Moreover, the knowledge hiding behavior has caused a loss of more than $31.5 billion among 500 companies (Babcock, 2004). Based on the adverse effects of knowledge hiding behavior, its antecedents need to be studied to identify what causes knowledge hiding behavior.

Recent studies suggest that knowledge hiding is greatly dependent on the leadership style (Jha and Varkkey, 2018; Khalid et al., 2018). Sometimes, knowledge hiding situation emerges when leaders become inactive and do not encourage learning and growth. Thus, it is important to have an active leader rather than a passive leader. Sharing of data improves the behavior of employees toward their tasks and toward creating innovativeness. Few previous literature studies identify passive leaders as the ones who display the actions that may result in delaying or avoiding decision-making, ignoring or being inattentive to workplace problems, and being unable to follow the expected standards of behavior while communicating with the team (DeRue et al., 2011). According to Blau (1964), distrust, interpersonal relationships, and social exchanges develop the knowledge hiding behavior of employees. Passive leaders avoid social exchanges; they are mostly lazy toward solving problems; they avoid making decisions; they do not take actions easily; and they are not active when required (Kelloway et al., 2005). Therefore, it can be said that passive leaders do not make decisions until the situation gets out of hand.

If we talk in terms of IT projects, there are many situations (uncertainties and complexities) where prompt responses are needed (Shenhar, 2001), and where knowledge sharing becomes essential. In those situations, knowledge hiding behavior, which is very common, becomes destructive for project-based organizations. In most of the projects, very limited information is available at the beginning, so an active leader can move the project smoothly by proactively figuring out situations, identifying possibilities and understanding complexities. An active leader, contrary to a passive leader, can provoke others to share more and more knowledge, thus lessening the intentions to hide knowledge. Because the available information related to the projects is usually ambiguous at the beginning, so the team needs capabilities to create an influencing and lively environment (Sauser et al., 2009; Patil and Suresh, 2019). Thus, in such cases, leaders should behave professionally so that the organization can deal with ambiguities and uncertainties, which would have become impossible to achieve under a passive leader. Therefore, it can be said that a passive leader can promote knowledge hiding behavior.

Under a passive leader, employees may not feel the need to take part in things actively, thus, failing to provide important knowledge to the other team members. Therefore, poor communication may result in knowledge hiding behavior. Active leaders not only solve problems but also perform their tasks honestly and warn the employees about consequences. Furthermore, active leaders always get themselves involved in the situation to meet organizational objectives and promote knowledge sharing instead of knowledge hiding (Tang et al., 2015). Such leadership traits are not found in passive leaders as passive leaders are not prone to actively solve problems.

Based on the above arguments, we propose that:

*H1: Passive leadership is positively related to knowledge hiding behavior*.

### The Moderating Role of Creative Self-Efficacy Between Passive Leadership and Knowledge Hiding

Creative self-efficacy is a situation wherein individuals suggest that they have the ability to produce creative outcomes (Tierney and Farmer, 2002); it is a major element to inculcate creative performance (Tierney and Farmer, 2002, 2004, 2011; Carmeli and Schaubroeck, 2007; Gong et al., 2009; Richter et al., 2012; Christensen-Salem et al., 2020). Self-efficacy affects the motivation level of individuals; it impacts the way people believe, think, and feel (Bandura, 1986, p. 25). The negative leadership style (passive leadership) can be reduced by incorporating the personal resource of creative self-efficacy as such individuals have the ability to produce creative outcomes. To produce creative and successful projects, individuals with creative self-efficacy do not hold and hide knowledge intentionally. They put more focus on producing the outcomes and eliminating the effects of passive leadership. Thus, this important issue of knowledge hiding may be avoided if the creative self-efficacy of an employee is high. Likewise, it is also reported that, while looking at the upcoming projects at the workplace, an employee with high self-efficacy will have more confidence which they will be able to utilize by becoming creative and by focusing on their project tasks (Black et al., 2019). Moreover, creative self-efficacy can also be built and improved (Chang et al., 2019). So, IT project organizations should focus on developing this important resource among employees to mitigate the negative effects of leadership style. On the contrary, creative self-efficacy is found to have no relation with passive leadership behavior (Parker et al., 2006). Thus, even in the case of a passive leader who is inactive and less supportive toward creativity and of high creative self-efficacy of an employee, then the employee would not get affected by the passive leadership behavior. According to the conservation of resources theory, employees utilize the key psychological resources (e.g., creative self-efficacy) to cope with the stressors (e.g., passive leadership) (Hobfoll, 2011). Therefore, the positive resource of employees is very important for organizations (Strauss et al., 2009). This is especially the case with IT projects as they are ever emerging and always need something creative to be successful.

In other words, the personal resources of an individual can lessen the effects of passive leadership. Creative self-efficacy may not let employees getting affected by passive leadership, and employees, in return, will not hide knowledge. Thus, we propose that employees with creative self-efficacy will not be influenced by the passive leadership behavior and will not show knowledge hiding behavior. Therefore, it can be said that there is a big margin to conduct research in this area as previously no other study has explored passive leadership and knowledge hiding through creative self-efficacy.

*H2: Creative self-efficacy acts as a moderator between passive leadership and knowledge hiding*.

## Methodology

To test the stated objectives, data were collected from the IT project-based organizations. Multiple project-based organizations of Rawalpindi and Islamabad were approached. After explaining the purpose of the study, consent was achieved from employees to collect the data. Respondents were ensured that their identity will be kept confidential. Questionnaires were distributed in the English language. A purposive sampling technique was used to collect the data. Maxwell (1996) defined purposive sampling as a type of sampling in which particular settings, persons, or events are deliberately selected to get the important information that only they can provide and which cannot be obtained from other sources. Moreover, we chose a purposive sampling technique for various reasons: first, the selected respondents should be engaged in active projects, second, the respondents should have previous experience of projects, and finally, the selected respondents should be willing to volunteer for the research. To minimize the common method biased effect, data were collected in time lag as recommended by Podsakoff et al. (2003). We collected the data at three different points with a time lag of 3 weeks in each interval as done by previous researchers in similar contexts (Javed et al., 2017; Khan et al., 2020; Imam and Zaheer, 2021). In time 1 (T1), the data related to an independent (passive leadership) variable and demographics (age, gender, education, and experience) were collected. In time 2 (T2), the data related to a moderator (creative self-efficacy) were collected. In time 3 (T3), the data related to a dependent variable (knowledge hiding) were collected. In T1, 500 questionnaires were distributed, out of which 437 respondents gave their responses. About seven responses were discarded due to missing values, so 430 were left for further analysis. In T2, 430 questionnaires were distributed among the same respondents as that of T1, out of which, 387 questionnaires were returned, and 11 responses were discarded due to the missing values. Finally, in T3, 376 questionnaires were distributed, out of which 350 were returned. A secret code (grandfather's name) was used to match the T1, T2, and T3 respondents. After matching T1, T2, and T3 responses, 323 of them were considered for the analysis. Demographics of the study show that the majority of the respondents were men, i.e., 73%. Most of the respondents were between the age of 31 and 35, i.e., 58%. Similarly, most of the respondents, i.e., 61%, had a Master's degree. Similarly, 58% of the respondents had <5 years of experience.

### Measurements

#### Passive Leadership

We used a 5-item scale developed by Barling and Frone (2017) to measure passive leadership. Sample items included “Your supervisor delays taking action until problems become serious” and “Your supervisor avoids getting involved when important issues arise.”

#### Knowledge Hiding

Knowledge hiding was measured on a 12-item scale developed by Connelly et al. (2012). Sample items included “I offered him/her some other information instead of what he/she really wanted” and “I said that I did not know, even though I did.”

#### Creative Self-Efficacy

Creative self-efficacy was measured using a 9-item scale developed by Carmeli and Schaubroeck (2007). Sample items included “When facing difficult tasks, I am certain that I will accomplish them creatively,” and “I will be able to overcome many challenges creatively.”

## Results

### Measurement Model

In the current study, we performed the partial least squares (PLS) algorithm with 300 iterations to confirm the reliability and validity of the measurement model. To assess the reliability, we used composite reliability (CR) and rho_A with a cut-off value of 0.7. As shown in Table 1, all the constructs exceeded the recommended values, hence indicating acceptable reliability. The convergent validity of the constructs was assessed using CR and average variance extracted (AVE). As shown in Table 2, CR values are in the range between 0.87 and 0.90, which exceeded the recommended value of 0.70 (Hair et al., 2013). Similarly, the AVE values of all the constructs also exceeded the recommended value of 0.50 (Hair et al., 2013). In this way, the convergent validity of all the constructs was confirmed. Furthermore, discriminant validity was confirmed using the heterotrait-monotrait (HTMT) ratio of correlations (Henseler et al., 2015). As shown in Table 2, all the construct values are <0.90 (Kline, 2011). Moreover, as presented in Table 2, Fornell and Larcker (1981) criterion was adopted wherein the diagonal values (the square root of AVEs) are higher than the correlation coefficients. In this way, the discriminant validity of all the constructs was confirmed.

**Table 1:** Validity and reliability of constructs.

	**Loading**	**Alpha reliability**	**AVE**	**CR**	**rho_A**
**Passive leadership**		0.87	0.66	0.90	0.87
PL1	0.76				
PL2	0.83				
PL3	0.84				
PL4	0.84				
PL5	0.78				
**Creative self-efficacy**		0.81	0.59	0.87	0.81
CSE1	0.72				
CSE2	0.76				
CSE3	0.76				
CSE4	0.79				
CSE5	0.74				
CSE6	0.75				
CSE7	0.73				
CSE8	0.74				
**Knowledge hiding**		0.93	0.58	0.94	0.93
KH1	0.76				
KH2	0.78				
KH3	0.76				
KH4	0.75				
KH5	0.77				
KH6	0.76				
KH7	0.75				
KH8	0.76				
KH9	0.78				
KH10	0.76				
KH11	0.73				
KH12	0.77				

**Table 2:** Discriminant validity.

**S no**.	**Variables**	**1**	**2**	**3**
**Fornell and Larcker's (** **1981** **) procedure**				
1	Passive leadership	**0.81**		
2	Creative self-efficacy	−0.21	**0.75**	
3	Knowledge hiding	0.42	−0.28	**0.76**
**Heterotrait-Monotrait (HTMT)**				
1	Passive leadership			
2	Creative self-efficacy	0.31		
3	Knowledge hiding	0.25	0.46	

*Off-diagonal bold represents the square root of average variance extracted (AVE)*.

Finally, a confirmatory factor analysis (CFA) was used to assess the fitness of the three-factor model (passive leadership, creative self-efficacy, and knowledge hiding). Numerous researchers have already suggested different fit indicators [chi-squared, comparative fit index (CFI), incremental fit index (IFI), Tucker–Lewis index (TLI), and root mean square error of approximation (RMSEA)] to confirm the validity of the model (Dunn et al., 1993; Kelloway, 1998; Hu and Bentler, 1999; Kline, 2005). Therefore, this study showed excellent fitness of the model as per the suggestions given by the previous researchers, i.e., chi-square (χ^2^/*df*) = 1.99, CFI = 0.94, TLI = 0.93, IFI = 0.94, and RMSEA = 0.03. Moreover, we compared the baseline three-factor model with that of the one-factor model (combined all variables into one factor); the one-factor model showed the worst results as compared to the four-factor model. The results are presented in Table 3. Thus, based on the CFA results, we concluded that the model used for this study is a valid model for further analyses. Finally, we tested the common method bias by using Harman's single-factor method; according to Podsakoff et al. (2003), the recommended value should be <50%. Similarly, the value of the current study model was 32%, thus, indicating the absence of the common method bias.

**Table 3:** Confirmatory factor analysis (CFA).

**Models**	**Factors**	**χ^2^**	**DF**	**RMSEA**	**IFI**	**TLI**	**CFI**
Hypothesized model	Three factors model	513.59^**^	257	0.03	0.94	0.93	0.94
All in one factor	Single factor model	1,733.34^**^	374	0.11	0.58	0.56	0.58

*N = 323*;

** *p < 0.01*.

### Correlation Analysis

Table 4 shows SDs, means, and correlations among the variables. Passive leadership was positively correlated with knowledge hiding (*r* = 0.41, *p* < 0.01), and negatively correlated with creative self-efficacy (*r* = −0.21, *p* < 0.01). Moreover, creative self-efficacy was negatively correlated with knowledge hiding (*r* = −0.27, *p* < 0.01).

**Table 4:** Correlation.

**S.no**	**Variables**	**Mean**	**SD**	**1**	**2**	**3**
1	Passive leadership	3.24	0.89			
2	Creative self-efficacy	3.80	0.82	−0.21^**^		
3	Knowledge hiding	3.19	0.83	0.41^**^	−0.27^**^	

*N = 323*;

** *p < 0.01*.

### Hypotheses Testing

#### Direct Hypothesis

The first hypothesis of this study suggested that passive leadership behavior positively influences the knowledge hiding behavior of employees. We tested this hypothesis in PLS, and the results showed that passive leadership indeed positively influences the knowledge hiding behavior of employees (β = 0.36, *p* < 0.001). The results are displayed in Table 5.

**Table 5:** Hypotheses testing.

**Hypotheses**	**Beta**	***T* Values**	***p-*Value**	**Decision**
Direct effects				
PL → KH	0.36	6.473	0.001	Supported
Interaction effect				
PL×CSE → KH	−0.22	3.417	0.001	Supported

#### Moderation Analysis

The current study also hypothesized that creative self-efficacy would have a moderating effect on the relationship between passive leadership and knowledge hiding. The study employed PLS to analyze a moderator relationship. Henseler and Fassott (2010) suggested that PLS provides more accurate estimates of the moderator effects by taking into account the errors in the estimated relationships, thus improving the validation of the theory. As shown in the results in Table 5, the interaction effect of creative self-efficacy and passive leadership was negative and significant for knowledge hiding (β = −0.21, *p* < 0.001). Thus, Hypothesis 2 of this study is also proved to be true.

## Discussion

### Passive Leadership Is Negatively Related to Knowledge Hiding

As shown by the findings of this study, passive leadership is negatively related to knowledge hiding. Therefore, it can be said that passive leadership is one of the causes due to which people hide knowledge. If a passive leader remains inactive by not pointing out errors and mistakes, and by refraining from correcting others, then it may damage the organization. By doing so, passive leaders discourage others from spreading and sharing knowledge. This will not only create negative consequences for the employees of the company but also for the overall team and the project. Studies identify that bad leadership style and behavior show disengagement toward employees and work. Furthermore, passive leaders do not take action timely (Barling and Frone, 2017). So, it is important to say that every project needs a kind of leader who can share knowledge continuously. Moreover, according to Arain et al. (2019), a negative leadership style greatly influences the knowledge hiding behavior of individuals because the behavior of employees is influenced by top-down knowledge sharing behavior. Therefore, the inactive and passive behavior of a leader will create a huge impact on employees. As a result, employees will not bother to share the necessary information because of the top-down inactiveness and knowledge hiding behavior.

### Creative Self-Efficacy Acts as a Moderator Between Passive Leadership and Knowledge Hiding

The second objective of this study highlights the role of creative self-efficacy as a moderator between passive leadership and knowledge hiding. In the past two decades, ample research has been conducted to identify the positive impact of knowledge sharing on individuals, teams, and organizational productivity and performance (Quigley et al., 2007). Research has also been done to study team creativity and innovation, and the innovative capabilities of firms (Podrug et al., 2017). However, knowledge hiding behavior has not yet been studied considering the abovementioned variables. Previous studies indicate that the knowledge of individuals regarding a task is a determinant of creative self-efficacy (Amabile, 1983; Gist and Mitchell, 1992; Tierney and Farmer, 2002). Previous research has also proven that creative self-efficacy is influenced by leadership and predicts the creativity of employees (Zheng and Liu, 2017). However, in the previous studies, contradictory results were found wherein negative leadership, i.e., abusive leadership is seen to reduce the creative self-efficacy of individuals (Atamba et al., 2020). On the contrary, our results reveal that creative self-efficacy moderates the relation between passive leadership and knowledge hiding. A person with creative self-efficacy will not only get affected by a passive leader but will also tend to share knowledge with others. This is because self-efficacy and other personal resources result in competitiveness, achievement striving, and pro-social motivation, thereby reducing knowledge hiding behavior (Hernaus et al., 2019).

### Theoretical Implication

Although we know about the consequences of knowledge hiding, very less has been evidenced about the antecedents of knowledge hiding. Using the theory of planned behavior, this study contributes specifically to the project management literature by identifying the impact of passive leadership on the knowledge hiding behavior of employees. The Inactiveness of a passive leader impacts the knowledge hiding behavior of employees. Moreover, there are many moderating factors between passive leadership and knowledge hiding. Therefore, this study helps to show that employees with high creative self-efficacy may lessen the negative impact of passive leadership and knowledge hiding. A person with creative self-efficacy will not get bothered by a negative passive leadership style. Creative self-efficacy of individuals will let them collaborate and work with other team members without hiding knowledge and with an urge to work together to learn and create something new. Thus, the smooth functioning of IT organizations may be achieved by discouraging the passive leadership style and by preferring employees with creative self-efficacy.

### Practical Implication

This study comes with several practical contributions for project-based organizations. IT industry cannot run with passive and knowledge hiding behavior because of the rigorously changing demands of the marketplace. The study also addresses why Pakistan is lagging in innovative IT projects. Knowledge needed for innovation is being affected by passive leadership behavior. Thus, active and professional leaders are required in today's changing world. Moreover, this study provides evidence for the practitioners that if employees have strong personal resources, passive leadership will not affect their behavior. One of the personal resources that this study highlighted is creative self-efficacy, which lessens the negative effect of passive leadership. The study throws light on a very important aspect that high creative self-efficacy of an employee is important in scenarios where there is no leadership (passive leadership). Hence, this study addresses many unanswered questions, including the influence of creative self-efficacy on passive leadership and knowledge hiding.

### Limitations and Future Directions

This study only highlights the antecedents of knowledge hiding in projects, which limit the application of study in other sectors. In the future, data can be collected from other sectors to generalize the results of the study. Moreover, this study only provides the missing link between passive leadership and knowledge hiding; there are still many underlying factors that may influence knowledge hiding, e.g., insecurity, motivation, organizational structure, etc. Similarly, this study provides suggestions that how a personal resource, i.e., creative self-efficacy, mitigates the effect of negative leadership style and knowledge hiding practices. There are many other possible personal resources, which can moderate this relationship, and therefore are needed to be studied in future.

## Data Availability Statement

The original contributions presented in the study are included in the article/Supplementary Materials, further inquiries can be directed to the corresponding author/s.

## Ethics Statement

The studies involving human participants were reviewed and approved by CUST. The patients/participants provided their written informed consent to participate in this study.

## Author Contributions

All authors listed have made a substantial, direct and intellectual contribution to the work, and approved it for publication.

## Funding

This work was supported by Ministry of Higher Education Malaysia for Fundamental Research Grant Scheme with Project Code: FRGS/1/2020/SS0/USM/02/30.

## Conflict of Interest

The authors declare that the research was conducted in the absence of any commercial or financial relationships that could be construed as a potential conflict of interest.

## Publisher's Note

All claims expressed in this article are solely those of the authors and do not necessarily represent those of their affiliated organizations, or those of the publisher, the editors and the reviewers. Any product that may be evaluated in this article, or claim that may be made by its manufacturer, is not guaranteed or endorsed by the publisher.
