# Supplementary material for: What Makes People Hide Knowledge? Influence of Passive Leadership and Creative Self-Efficacy
Source: Front Psychol. 2021 Oct 8;12:740880. doi: 10.3389/fpsyg.2021.740880 (PMC8531077; doi:10.3389/fpsyg.2021.740880)
Supplement: Supplementary file 1 [file Data_Sheet_1.docx]

**Appendix**

**Cross-loading**

| **Constructs** | **PL** | **CSE** | **KH** |
| --- | --- | --- | --- |
| PL1 | .76 | .41 | .22 |
| PL2 | .83 | .39 | .25 |
| PL3 | .84 | .43 | .34 |
| PL4 | .84 | .45 | .39 |
| PL5 | .78 | .51 | .41 |
| CSE1 | .32 | .72 | .57 |
| CSE2 | .32 | .76 | .61 |
| CSE3 | .34 | .76 | .62 |
| CSE4 | .35 | .79 | .60 |
| CSE5 | .29 | .74 | .47 |
| CSE6 | .31 | .75 | .42 |
| CSE7 | .32 | .73 | .51 |
| CSE8 | .31 | .74 | .42 |
| KH1 | .53 | .42 | .76 |
| KH2 | .49 | .41 | .78 |
| KH3 | .55 | .41 | .76 |
| KH4 | .46 | .43 | .75 |
| KH5 | .43 | .39 | .77 |
| KH6 | .45 | .36 | .76 |
| KH7 | .46 | .47 | .75 |
| KH8 | .42 | .43 | .76 |
| KH9 | .41 | .41 | .78 |
| KH10 | .51 | .33 | .76 |
| KH11 | .52 | .35 | .73 |
| KH12 | .49 | .31 | .77 |

**Abbreviations:** PL, passive leadership; CSE, creative self-efficacy; KH, knowledge hiding.
